# Citron Juice

**Published:** 1879-12

**Authors:** 


Citron Juice in Chronic Enlargement of the Tonsils. M. de Saint-Germain paints chronically enlarged tonsils twice daily with citron juice with good effect, curing his cases generally within a fortnight.—*Jour. des Sci. Med. Louvain.*

